# Electrophysiological Correlates of Romantic Love: A Review of EEG and ERP Studies with Beloved-Related Stimuli

**DOI:** 10.3390/brainsci12050551

**Published:** 2022-04-26

**Authors:** Sandra J. E. Langeslag

**Affiliations:** Department of Psychological Sciences, University of Missouri—St. Louis, One University Boulevard, 417 Stadler Hall, St. Louis, MO 63121-4499, USA; langeslags@umsl.edu; Tel.: +1-314-516-5395; Fax: +1-314-516-5392

**Keywords:** romantic love, romantic relationships, love, event-related potential (ERP), late positive potential (LPP), early posterior negativity (EPN), P3, P300, electroencephalography (EEG), brain, attention

## Abstract

Science is starting to unravel the neural basis of romantic love. The goal of this literature review was to identify and interpret the electrophysiological correlates of romantic love. Electroencephalography (EEG) and event-related potential (ERP) studies with a design that elicits romantic love feelings were included. The methods of previous EEG studies are too heterogeneous to draw conclusions. Multiple ERP studies, however, have shown that beloved stimuli elicit an enhanced late positive potential (LPP/P3/P300), which is not due to familiarity, positive valence, or objective beauty. This effect occurs in Western and Eastern cultures and for pictorial and verbal information, and results from bottom-up rather than top-down factors. Studies have also shown that beloved stimuli elicit an early posterior negativity (EPN), which also does not seem to be due to familiarity or positive valence. Data on earlier ERP components (P1, N1, P2, N170/VPP, N2) is scarce and mixed. Of course, the enhanced LPP and EPN are not specific to romantic love. Instead, they suggest that the beloved captures early attention, within 200–300 ms after stimulus onset that is relatively resource-independent, and subsequently receives sustained motivated attention. Future research would benefit from employing cognitive tasks and testing participants who are in love regardless of relationship status.

## 1. Introduction

Research on romantic love is important because romantic love pertains to virtually everyone [1,2]. When people fall in love, it has a great impact on their lives [3]. Romantic love obviously has positive effects on people and society. Infatuation is associated with positive emotions such as euphoria, and romantic relationships enhance well-being [4,5]. What is often overlooked, though, is that romantic love has negative effects on people and society as well. For example, romantic love is associated with several negative emotions, such as anxiety, jealousy, grief, and shame [6,7,8], and dysfunctional romantic relationships and romantic break-ups are associated with a decrease in well-being and with depression [9,10,11]. Romantic love plays a role in several mental disorders, including sexual dysfunctions, paraphilic disorders, and erotomanic and jealous delusional disorders [12], as well as in suicidal behaviors [13]. Finally, romantic love is associated with various forms of criminal behavior, including stalking, domestic violence, and homicide [14,15,16,17]. It is clear that the high prevalence of romantic love combined with its positive and negative impact on individuals and society cause a critical demand for fundamental and applied scientific research on romantic love.

### 1.1. Neural Basis of Romantic Love

Thanks to recent advances in neuroimaging, we are beginning to unravel the neural basis of romantic love, including the neurotransmitters/hormones and brain regions/networks involved. Studies have shown that oxytocin, vasopressin, cortisol, serotonin, dopamine, follicle-stimulating hormone, and testosterone are involved in romantic love [18,19,20,21,22]. In addition, the caudate, putamen, ventral tegmental area, insula, amygdala, cingulate cortex, globus pallidus, substantia nigra, raphe nucleus, cerebellum, nucleus accumbens, thalamus, and various parts of the cortex have been shown to play a role in romantic love [23,24,25,26,27,28,29].

Other aspects of the neural basis of romantic love have been studied using electrophysiological methods such as electroencephalography (EEG) and event-related potentials (ERPs). The research question of this review is: What are the electrophysiological correlates of romantic love and what do they tell us? Of course, any aspects of the EEG signal or any ERP components that are affected by romantic love are not ‘love signals’ or ‘love components’, just like there are no ‘love neurotransmitters/hormones’ or ‘love brain regions/networks’. Instead, any effects of romantic love on EEG signals and/or ERP components reveal information about the cognitive processes affected by romantic love. For this literature review, PubMed, PsycInfo, and Google Scholar were searched for articles up to March 2022 using the following search terms: (romantic) love, event-related potential(s), ERP(s), electroencephalogram/graphy, and EEG.

One approach to studying the electrophysiological correlates of romantic love has been to present participants who are not necessarily in love with words like “love” and “kiss”, sentences describing romantic scenarios, or pictures/videos that depict strangers in romantic situations [30,31,32,33,34,35]. However, although those stimuli may elicit positive affect, they are unlikely to elicit feelings of romantic love. Another approach has been to compare participants who are in love (or in a relationship) with participants who are not in love (or in a relationship) on some task that is expected to elicit love feelings in the in-love participants. A final approach has been to present participants who are in love (or in a relationship) with stimuli related to their beloved/partner and control stimuli, and previous research has shown that beloved stimuli do indeed elicit feelings of romantic love [36,37,38]. Published electrophysiological studies that used a task and/or stimuli that can be expected to elicit love feelings were included in this narrative exhaustive review.

### 1.2. Valence and Familiarity

Studies using face pictures of the beloved have often used pictures of family members, friends, celebrities, babies, and/or strangers as control stimuli, which controls for general face perception processes. Two potential confounding factors when comparing beloved-related stimuli with control stimuli are valence and familiarity. It is important to note that romantic love is not necessarily associated with positive valence. Unreciprocated love is associated with negative affect [5,8,38], and while reciprocated love is prototypically associated with positive affect, it can be associated with negative affect (e.g., jealousy or anxiety) as well [5]. Thus, people who are in love do not necessarily feel pleasant, and beloved stimuli do not necessarily elicit more positive affect than family or friend stimuli. In addition, the beloved is not necessarily more familiar than family members or friends. There are various ways to operationalize familiarity, including frequency of interactions, depth of the interactions, time since last interaction, and/or time since first interaction. New beloveds in particular may be less familiar than family members or friends. Thankfully, the inclusion of celebrity, baby, and/or stranger stimuli in some studies allows for drawing conclusions about the potentially confounding effects of valence and/or familiarity, as discussed below.

## 2. Electrophysiological Studies on Romantic Love

Two types of central electrophysiological measures are EEG and ERP. Both of these measures involve attaching electrodes to the participant’s scalp and measuring the voltage differences on the scalp caused by the electric current in neurons. These two methods have a few limitations. First, they only measure cortical activity of open field populations of neurons (in which the neurons have similar orientations so that their electrical currents add up instead of cancel out) that are oriented perpendicular to the scalp. Second, they measure postsynaptic potentials instead of action potentials, as those are too brief. Third, the spatial resolution is poor because of the inverse problem. The main advantage of both of these methods, however, is that they are a direct measure of neuronal activity. Other advantages of EEG and ERP are that they are relatively affordable and non-invasive.

### 2.1. Electroencephalogram (EEG)

The EEG can be recorded while the participant is at rest, perceiving certain stimuli, and/or performing a certain task. In an early study [39], participants of unknown love status engaged in six different tasks, including mental imagery of a past, positive time of being in love with and without sexual imagery. The number of fractional dimensions was computed as a measure of EEG signal complexity. Both mental imagery tasks were associated with higher fractional dimensions than the other four visual, verbal, and sensory tasks, especially at frontal electrodes. In addition, the imagery task with sexual imagery elicited higher fractional dimensions than the imagery task without sexual imagery. It is difficult to interpret these effects though, because it is uncertain if retrieving memories of past instances of romantic love elicits feelings of romantic love in people who are currently not in love, and subjective measures of romantic love feelings were not collected. In another study [39], participants who were and were not in love were compared while imagining a positive scene with the beloved partner, a negative scene (jealousy), and a neutral scene. It was not clarified what the mental imagery of “the beloved partner” [39] (p. 133) entailed in the participants who were not in love. No differences in the number of fractional dimensions were observed between the two groups or the different imagery conditions. Thus, if anything, higher EEG signal complexity seems due more to mental imagery processes than to romantic love feelings. (It should also be noted that the first author of this article [39] was found guilty of scientific misconduct in some of his other research conducted in 2013 and 2014 [40].)

In more recent EEG studies, data have been analyzed using a Fast Fourier Transformation, which reveals the power of several frequency bands, such as delta (~1–4 Hz), theta (~4–8 Hz), alpha (~8–13 Hz), beta (~13–20 Hz), and gamma (~>20 Hz). These bands reflect neural oscillations of different frequencies that occur, depending on the state of the participant. For example, the power of the lower delta and theta bands is high during sleep, the alpha power is high during relaxation, and the power of beta and gamma bands is high during cognitive processing [41]. In one study [42], female participants who were in a romantic relationship viewed partner, friend, and stranger pictures as well as an empty gray square. No differences between the different face pictures were observed in the theta, alpha, beta, or gamma bands. The partner pictures did elicit higher delta (0.5–3 Hz) power than the friend and stranger pictures, and this effect was largest at the frontal electrodes. Even though love intensity or a similar variable was not measured, this frontal increase in delta power was interpreted as reflecting the affection felt towards the romantic partner.

Recent EEG studies have also analyzed so-called frontal alpha asymmetry. Frontal alpha asymmetry reflects the balance between left and right frontal brain activation, with greater left than right frontal activation indicating a motivation to approach and greater right than left frontal activation indicating a motivation to avoid [43]. Because alpha power is inversely related to brain activation and because romantic love could be expected to be associated with the motivation to approach the beloved, the hypothesis is that beloved stimuli would elicit greater right than left frontal alpha power. In one study [35], participants who were in love (and in a relationship) were compared with participants who were not in love (and not in a relationship). Participants who were in love listened to a self-selected song that reminded them of their beloved and wrote and talked about events related to their relationship to induce love feelings. Participants who were not in love listened to one of the songs selected by the in-love participants and wrote and talked about the romantic relationship of a friend. While listening to the song (but not during rest), the participants who were in love showed higher alpha power over the right than the left occipital scalp, whereas the participants who were not in love showed higher alpha power over the left than the right occipital scalp. Although the lateralization of the alpha asymmetry in the participants who were in love was in line with the hypothesis, the effect occurred at occipital rather than frontal electrodes, which limits the interpretation in terms of approach and avoidance motivation. Instead, the authors interpreted their findings as reflecting internally focused attention in the participants who were in love and boredom/disengagement in the participants who were not in love.

To summarize, mental imagery related to romantic love caused increased EEG signal complexity compared to sensory tasks [39], but this is probably more a reflection of mental imagery processes than romantic love feelings. In addition, partner pictures elicited higher frontal delta power than friend or stranger pictures [42], and listening to love-related songs elicited higher alpha power over the right than the left occipital scalp in participants who were in love, but the reverse pattern in participants who were not in love [35].

### 2.2. Event-Related Potentials (ERPs)

ERPs reflect brain activity time-locked to events, such as stimuli and/or tasks. After recording the voltage differences on the scalp caused by the electric current in neurons (i.e., the EEG), ERPs are extracted by averaging those parts of the EEG that occur during the stimuli/tasks. This averages out neural oscillations and noise and leaves the electrical activity elicited by the stimulus and/or task. The main advantages of ERPs are that they have a temporal resolution in the order of milliseconds and that they provide information about cognitive processes [44,45]. The ERP consists of different components, each of which has a certain polarity, latency, and scalp topography, and reflects a cognitive process. Although ERP component names are descriptive (e.g., the N1 is the first negative peak; the P300 is a positive peak around 300 ms after stimulus onset), different names are sometimes used for the same component, and vice versa. In this review, study results will be described using the names used in the original publication. Different ERP components are discussed below, not in the order in which they appear in the ERP, but in the order of descending empirical data availability.

#### 2.2.1. Late Positive Potential (LPP)

One ERP component is the late positive potential (LPP), which consists of multiple and overlapping positivities (including the P3/P300) over the posterior scalp, beginning around 300 ms after stimulus onset [46]. Because the LPP is larger for positive and negative than neutral stimuli and can last for seconds [47], it is thought to reflect sustained motivated attention [48].

##### Passive Viewing Paradigms

**Figure 1:**
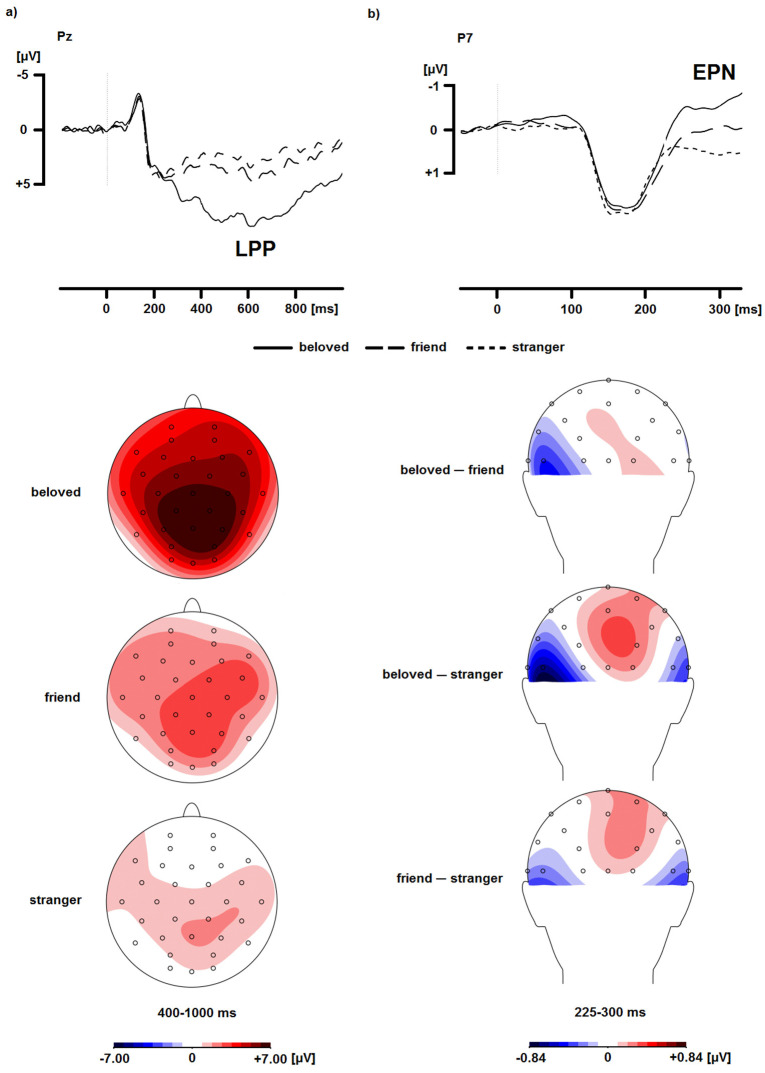
Examples of the enhanced LPP (**panel a**) and the EPN (**panel b**) in response to the beloved, adapted from [50]. As is common in ERP figures, positive is plotted downward. Because the EPN is a relative difference, scalp topography effects are depicted.

Multiple ERP studies on romantic love have employed a passive viewing paradigm, although some studies included a simple task to keep participants engaged. In a study with participants who were in a romantic relationship [49], partner pictures elicited a larger P3 at centroparietal midline electrodes as well as a larger LPP at centroparietal midline electrodes than celebrity and stranger pictures. Neither the P3 nor the LPP amplitude differed significantly between celebrities and strangers, suggesting that the enhanced LPP in response to the partner was not due to familiarity. In another study with participants who were in love [50], beloved pictures elicited a larger LPP than friend and stranger pictures across the scalp (yet mostly at centroparietal electrodes). The difference in LPP amplitude between friend and stranger pictures did not reach significance, suggesting that the enhanced LPP in response to the beloved is not due to familiarity or positive valence. In another study with participants who were in love [51], beloved pictures elicited a larger LPP than friends or objectively beautiful strangers across the scalp (yet mostly at centroparietal electrodes), which suggests that the enhanced attention for the beloved is not due to objective beauty. See Figure 1a for an example of the effect of beloved stimuli on the LPP.

In another study [52], participants were members of the Chinese Mosuo tribe, which has two marriage styles: intermarriage and walking marriage. In an intermarriage, the married couple and their children form the family unit. In a walking marriage, in contrast, siblings and their children form the family unit, and lovers only meet overnight. Spouse/lover pictures elicited a larger P300 than siblings pictures (albeit at posterior electrodes in intermarriage participants and at left anterior electrodes in walking marriage participants), which shows that the enhanced attention for the beloved generalizes to Eastern cultures. In another study with participants who were in love [53], the LPP was largest in response to beloved-related words/phrases, intermediate in response to friend-related words/phrases, and smallest in response to control words/phrases at parietal electrodes, which shows that the enhanced attention for the beloved generalizes to verbal information.

One study that seems contradictory is one in which boyfriend pictures elicited a smaller P3 than father pictures in female participants who were in a relationship, although the frontocentral scalp topography of this effect suggests that it is different from the typical centroparietal P3 effect. In addition, the LPP was not significantly different between boyfriend and father pictures [54]. It may be that the LPP difference between boyfriend and father is smaller than the difference between partner/beloved/spouse/lover and celebrity/friend/sibling, so that more trials and/or participants would be needed to observe a significant effect on the LPP amplitude.

To summarize, the large majority of studies have shown that an enhanced LPP/P3/P300 in response to beloved-related information is an ERP correlate of romantic love. Because the LPP reflects motivated attention [48], this implies that romantic love is accompanied by enhanced motivated attention for the beloved. The existing studies suggest that this enhanced motivated attention for the beloved is not due to familiarity, positive valence, or objective beauty, and that it generalizes to Eastern cultures and verbal information.

##### Task Paradigms

In some studies, participants did not just passively view stimuli, they completed a cognitive task instead. Employing a task greatly aids in the interpretation of love effects on ERP components. In one study, participants who were in love performed an oddball task with infrequent beloved and friend pictures [55]. In some blocks, participants were instructed to count the beloved pictures, making those the target and the friend pictures the distractor, and vice versa in other blocks. Target pictures elicited a larger P3 across the scalp (but most pronounced at the midline parietal electrode) than distractor pictures, regardless of the person depicted, which confirms the interpretation of the P3 as reflecting attention. Additionally, beloved pictures elicited a larger P3 than friend pictures across the scalp, which shows that people pay more attention to their beloved than their friend. Importantly, this love effect on the P3 occurred regardless of the target/distractor status of the pictures, which implies that the enhanced attention for the beloved occurred regardless of the instruction to pay attention to the beloved or not. In two other studies [56], participants who were in love performed short-term memory tasks with neutral shapes while task-irrelevant face pictures of the beloved, friend, and strangers were presented. Participants were informed that the faces were task-irrelevant (study 1) or were instructed to ignore them (study 2). Still, beloved pictures elicited a larger LPP than friend pictures across the scalp in both studies.

To summarize, three studies have shown that beloved pictures capture attention even when they are task-irrelevant and/or supposed to be ignored. Therefore, the enhanced LPP in response to beloved pictures does not seem the result of top-down factors (i.e., instructed (in)attention), but to bottom-up factors (e.g., emotional salience) instead.

#### 2.2.2. Early Posterior Negativity (EPN)

The early posterior negativity (EPN) is a relative negativity over the occipital scalp between 200–300 ms after stimulus onset. The EPN emerges as the difference between electrophysiological responses to experimental and control stimuli [48,57], which makes it an ERP effect rather than an ERP component. The EPN occurs for emotional (vs. neutral) stimuli that have evolutionary significance [58,59,60] and is thought to reflect early attentional capture that is relatively resource-independent [48].

Three studies have tested the effect of romantic love on the EPN. In the two studies in which task-irrelevant beloved, friend, and stranger pictures were presented during short-term memory tasks, an EPN occurred between 250–400 ms for beloved (vs. friend) pictures at lateral occipital (study 1) and lateral parietal (study 2) electrodes [56]. In another study with participants who were in love [50], an EPN occurred between 225–300 ms in response to beloved (vs. friend) pictures at a left parietal electrode in a rapid serial visual presentation task and at lateral parietal and lateral and midline occipital electrodes in a slower, passive viewing task. See Figure 1b for an example of the effect of romantic love on the EPN. Taken together, these studies suggest that an EPN for the beloved occurs with both fast and slow stimulus presentation. In three of these four analyses, there was no EPN for friend (vs. stranger) pictures [50,56], which suggests that the EPN for the beloved is not the result of familiarity or positive valence. The effect of beloved stimuli on the EPN suggests that the beloved captures early attention that is relatively resource-independent, which probably reflects the evolutionary significance of the beloved due to romantic love playing an important role in reproduction [61].

#### 2.2.3. Earlier ERP Components

Several of the above-mentioned studies have tested the effect of romantic love on other ERP components. The P1, N1, and P2 are early visual ERP components that occur between 100–200 ms [45]. There is no evidence that the P1 differs between task-irrelevant beloved, friend, and stranger pictures [56], that the N1 differs between boyfriend, father, stranger and baby pictures [54], or that the P2 differs between beloved, friend, and beautiful stranger pictures [51]. However, more data are needed.

The N170 is a negative peak around 170 ms over the lateral posterior scalp that reflects structural face encoding [62]. The frontocentral vertex positive potential (VPP) is a different manifestation of the same process [63]. Although there was no evidence that the N170 differs between boyfriend, father, stranger, and baby pictures [54] or between task-irrelevant beloved, friend, and stranger pictures [56], spouse/lover pictures elicited a larger VPP than sibling pictures in the Mosuo tribe sample [52]. Thus, the sparse evidence concerning the effect of romantic love on the N170/VPP is mixed.

The N2 is a negative deflection starting around 200 ms that reflects conflict detection [64]. The N2 was smaller in response to beloved than friend and stranger pictures over the right hemisphere [51], and the N250 was smaller in response to spouse/lover compared to sibling pictures at frontocentral electrodes [52]. In contrast, no difference in the N2 at midline electrodes was observed between boyfriend and father pictures [54]. Thus, the little data available are mixed, but if anything, the N2 seems reduced in response to the beloved.

## 3. Conclusions

The goal of this review was to identify and interpret the electrophysiological correlates of romantic love. This narrative exhaustive review included published electrophysiological studies that used a design that elicits romantic love feelings. The approach and scope of this literature review have some limitations and strengths. First, no meta-analysis was performed to estimate the size of the effect of beloved-related stimuli on the EEG signal or ERP components. Second, no unpublished studies were included, so publication bias was not assessed. A strength of this literature review is that it is the first to summarize existing findings on the electrophysiological correlates of romantic love, leading to recommendations and suggestions for future research that can help move the field forward.

### 3.1. EEG Correlates of Romantic Love

The four existing EEG studies are difficult to compare because they assessed different dependent EEG variables and used different types of stimuli/tasks. In two early studies [39], the higher EEG signal complexity associated with mental imagery related to romantic love seemed due more to mental imagery than to romantic love. Another EEG study suggested that partner pictures elicit increased frontal delta (0.5–3 Hz) power [42]. A final study found no support for the hypothesis that beloved stimuli would elicit greater right than left frontal alpha power. Instead, listening to love-related songs elicited higher alpha power over the right than the left occipital scalp in participants who were in love, but the reverse pattern in participants who were not in love [35]. Because of the scarcity of the data, these EEG correlates await replication in future studies.

### 3.2. ERP Correlates of Romantic Love

Multiple ERP studies have shown that the LPP (including the P3/P300) is enhanced in response to beloved stimuli. This effect does not result from familiarity, positive valence, or objective beauty and generalizes to Eastern cultures and verbal stimuli. Several studies have shown that an EPN occurs in response to the beloved and have suggested that that is not due to familiarity or positive valence. Thus, the ERP correlates of romantic love seem to be an enhanced LPP (including the P3/P300) and EPN in response to the beloved.

Of course, the enhanced LPP and EPN are not specific to romantic love, as they also occur for emotional stimuli in general [48,57]. To someone who is in love, their beloved is a highly emotional and arousing stimulus [50,54,65], which is in line with the idea that the LPP and EPN are modulated by stimulus arousal [48]. In other words, the LPP and EPN are not ‘love ERP components/effects’ by any means. Instead, the enhanced LPP and EPN for beloved stimuli reveal which cognitive processes are enhanced for beloved-related information. Both the LPP and EPN reflect attentional processes, albeit different ones. In line with the earlier latency, the EPN is thought to reflect early attentional capture that is relatively resource-independent [48]. In line with the later onset and longer duration, the LPP is thought to reflect sustained motivated attention [48]. Thus, the interpretation of the ERP correlates of romantic love is that the beloved captures early attention, within 200–300 ms after stimulus onset that is relatively resource-independent, and subsequently receives sustained motivated attention that can last for seconds.

This enhanced attention for the beloved resembles the enhanced attention for emotional information in general [66] and is in line with some behavioral data specific to romantic love. In an oddball task, false alarm rates (i.e., erroneously pressing a button in response to a distractor picture) were higher for beloved than friend pictures [23]. In another study, T2 detection in an attention blink task with faces was better when T2 was the partner compared to a friend or stranger, which correlated with love intensity as measured with the Passionate Love Scale [67]. The enhanced attention for the beloved also resembles the attention biases associated with mental disorders such as depression and anxiety [68,69], which raises questions about the adaptiveness of the enhanced attention for the beloved. While paying attention to the beloved may often be adaptive, it may become maladaptive when it leads to insufficient attention to other things (e.g., (home)work, family, friendships) or when the love is unreciprocated (e.g., after a break-up or resulting in stalking) [16,70,71]. Future research could focus on how to increase adaptive attention to the beloved and decrease maladaptive attention to the beloved.

The evidence regarding the earlier ERP components P1, N1, P2, N170/VPP, and N2 is scarcer and mixed; more research is needed to draw conclusions about whether the cognitive processes these components reflect are altered in response to the beloved. Other ERP components deserve attention as well. For example, it would be interesting to test whether romantic love affects ERP components/effects related to memory, such as old/new effects [53,72] and the early repetition effect (N250r) [73], or ERP components/effects related to conflict and reward processing, such as the error-related negativity (ERN) and feedback-related negativity (FRN) [74,75]. It would be helpful to use tasks known to elicit these components/effect as this aids in identification of the components/effects and interpretation of the effects of romantic love. In general, the romantic love ERP community would benefit from paying more attention to the scalp topography of ERP components/effects, to ensure correct identification of components/effects and the cognitive process they reflect and to improve comparability between studies.

### 3.3. Other Recommendations for Future Research

When studying the feeling of romantic love, there are some important design considerations. First, when using a romantic love induction task in participants who are in love and including participants who are not in love as control participants, it is important to ensure that the task makes sense for the participants who are not in love. In addition, when studying the feeling of romantic love, it is important to use tasks/stimuli that are known to elicit feelings of romantic love. Finally, it is important to note that not all romantic love happens within relationships and that people who are in a relationship are not necessarily in love. Thus, it may be more informative to test participants who are in love (regardless of relationship status) than participants who are in a romantic relationship (with unknown love status).
